# Cellular Basis for the Enhanced Efficacy of the Fms-Like Tyrosine Kinase 3 Ligand (FL) Adjuvanted VCG-Based *Chlamydia abortus* Vaccine

**DOI:** 10.3389/fimmu.2021.698737

**Published:** 2021-06-24

**Authors:** Shakyra Richardson, Fnu Medhavi, Tayhlor Tanner, Stephanie Lundy, Yusuf Omosun, Joseph U. Igietseme, Darin Carroll, Francis O. Eko

**Affiliations:** ^1^ Department of Microbiology, Biochemistry and Immunology, Morehouse School of Medicine, Atlanta, GA, United States; ^2^ National Center for Emerging Zoonotic and Infectious Diseases, Centers for Disease Control and Prevention (CDC), Atlanta, GA, United States

**Keywords:** *Chlamydia abortus*, Pmp18D, vaccine delivery, Flt3L, adjuvant

## Abstract

Efficacious vaccines are needed to control genital chlamydial diseases in humans and the veterinary industry. We previously reported a *C. abortus* (Cab) vaccine comprising recombinant *Vibrio cholerae* ghosts (rVCG) expressing the conserved and immunogenic N-terminal region of the Cab polymorphic membrane protein D (rVCG-Pmp18.1) protein that protected mice against intravaginal challenge. In this study, we investigated the immunomodulatory effect of the hematopoietic progenitor activator cytokine, Fms-like tyrosine kinase 3-ligand (FL) when co-administered with the rVCG-Pmp18.1 vaccine as a strategy to enhance the protective efficacy and the potential mechanism of immunomodulation. Groups of female C57BL/6J mice were immunized and boosted twice intranasally (IN) with rVCG-PmpD18.1 with and without FL or purified rPmp18.1 or rVCG-gD2 (antigen control) or PBS (medium) per mouse. The results revealed that co-administration of the vaccine with FL enhanced antigen-specific cellular and humoral immune responses and protected against live Cab genital infection. Comparative analysis of immune cell phenotypes infiltrating mucosal and systemic immune inductive tissue sites following immunization revealed that co-administration of rVCG-Pmp18.1 with FL significantly enhanced the number of macrophages, dendritic and NK cells, *γ*δ and NK T cells in the spleen (systemic) and iliac lymph nodes (ILN) draining the genital tract (mucosal) tissues compared to rVCG-Pmp18.1 alone. Furthermore, FL enhanced monocyte infiltration in the ILN, while CD19+ B cells and CD4+ T cells were enhanced in the spleen. These results indicate that the immunomodulatory effect of FL is associated with its ability to mobilize innate immune cells and subsequent activation of robust antigen-specific immune effectors in mucosal and systemic lymphoid tissues.

## Introduction

*Chlamydia abortus* is an obligate intracellular gram-negative bacterium and a major cause of placental infection in farm animals, including sheep resulting in Ovine Enzootic Abortion (OEA) (1). Infection is also found in goats, pigs and cattle leading to considerable economic losses in animal husbandry worldwide (1–4). It has been shown that natural infection of ewes does not result in apparent immediate clinical effect, the infection remaining latent until the animal becomes pregnant, after which the organism invades the placenta, multiplies, and eventually causes abortion (5). Oral inoculation or targeted administration of *C. abortus* over the tonsils of pregnant ewes has been shown to induce a placental infection (6, 7). Also, Gutierrez et al. (8) have induced placental infection following the oral administration of a high dose (5 x 10^9^ inclusion-forming units, IFU) of *C. abortus* prior to pregnancy, thus establishing latency. We recently found 10^7^ IFU of *C. abortus* strain AB7 caused tubal dilatation in mice after a single intranasal infection whereas intravaginal inoculation with 2 x 10^7^ IFU did not induce genital tract pathology (unpublished observation). These reports implicate the oral-nasal route as the natural port of entry for *C. abortus* in OEA. *C. abortus* infection also poses a zoonotic risk to pregnant women. Zoonotic infections are frequently asymptomatic and infected individuals are therefore often untreated leading to the development of complications, including severe septicemia with disseminated intravascular coagulation (DIC), resulting in spontaneous abortion of the fetus, preterm labor or stillbirth (9–11). Although antibiotics are effective against *Chlamydia*, most infections are asymptomatic and so many infected individuals do not seek treatment resulting in the onset of pathology being the first indication of an infection. It is therefore the considered scientific opinion that a vaccine capable of protecting against infection or even lessening severe disease would be the most effective approach for controlling these infections and the resulting complications (12, 13).

The currently available live attenuated *C. abortus* vaccines are based on the 1B strain and include, Enzovax^®^ and CEVAC Chlamydophila^®^. Although a single dose of each vaccine is effective, they are expensive, requiring microbe culture in tissue cells or embryonated eggs. They are thus labor-intensive, hazardous to produce, and challenging to manufacture in large quantities. Importantly, though these live attenuated 1B vaccines were initially thought to be safe and effective in preventing infection in sheep, they have been implicated in cases of abortion (14) prompting their discontinued use by farmers. The association of single nucleotide polymorphisms (SNPs) with the 1B vaccine strain in a recent study confirmed that this strain was not really attenuated and was being transmitted *via* vaccinated animals (15, 15). More recently, the 1B vaccine strain has been reported to produce placental pathology indistinguishable from wild type *C. abortus* infection (16). Besides, following vaccination, it is impossible to distinguish infected from vaccinated animals by serology alone (17), making it difficult to monitor vaccination practices. In addition, inactivated and DNA vaccines while promising in principle, have not been as effective as native antigen in protecting sheep against *C. abortus* (18) calling for alternative strategies to develop safe and effective vaccines.

The use of whole chlamydial agents as vaccine candidates has not been favorable due to the potential existence of immunopathogenic components as revealed in early human trials with inactivated whole chlamydial agents in which vaccinated individuals suffered exacerbated disease during subsequent infection (19, 20). Also, the recently developed genetic tools to generate stably attenuated and safe chlamydial vaccine strains are yet to be widely applied for generating attenuated chlamydial strains for human vaccine use (21). Thus, our current focus is to develop vaccines based on chlamydial subunit components. In addition to the chlamydial outer membrane protein, MOMP, several immunogenic proteins have been predicted that may serve as potential vaccine candidates. Among these is a unique family of proteins, the polymorphic membrane proteins (Pmps) (22). Genome sequencing of *C. abortus* has revealed the presence of 18 pmp genes as opposed to the 9 in *C. trachomatis* (23). The Pmps have been associated with virulence and resemble autotransporters of the type V secretion system (24, 25). In *C. trachomatis*, PmpD is a major protective antigen found on the surface of chlamydial elementary bodies (EBs) (24, 26, 27) and capable of generating neutralizing antibodies (28). This protein is evolutionarily conserved and involved in chlamydial attachment to host cells. Similarly, the Pmp18D of *C. abortus* is a highly conserved and immunogenic outer membrane protein that is expressed throughout the chlamydial developmental cycle making it a viable vaccine and diagnostic candidate.

Unfortunately, the choice of a subunit vaccine approach imposes certain design constraints, including the requirement for a delivery and adjuvant system that would effectively present antigens to the immune system and bolster protective immunity against *Chlamydia*. In this respect, the *Vibrio cholerae* ghost (VCG) vaccine delivery platform has been shown to be an effective delivery system for chlamydial vaccine antigens, eliciting antigen-specific immune responses and substantial protective immunity (29–32). Several adjuvants and immunomodulators have been employed to bolster the protective immune responses of a variety of chlamydial vaccine antigens (33–37). Targeting antigens to dendritic cells (DCs) is also important for inducing protective immunity against *Chlamydia* due to their proclivity for activating the Th1 immune response that is vital for chlamydial immunity (38–41). The Fms-like tyrosine kinase 3 ligand (Flt3L; FL) is a cytokine and growth factor, which binds to the fms-like tyrosine kinase receptor Flt3/Flk2 (CD135) to stimulate the proliferation and differentiation of several hematopoietic progenitors, including DCs (42, 43). Intranasal immunization of mice with FL and the non-typeable *Haemophilus influenzae* (NTHi) P6 protein increased dendritic cell numbers in the nasal-associated lymphoid tissue and enhanced antigen-specific long-term mucosal immune responses in the nasopharynx (44). Intramuscular immunization of mice with a recombinant rabies virus expressing mouse Flt3L enhanced DC maturation *in vitro* and *in vivo*, and significantly increased the induction of follicular helper T cells (45). A combination of FL and Granulocyte macrophage-colony stimulating factor (GM-CSF) was found to significantly increase splenic DC maturation and function (42) and elicited mucosal immunity to influenza in aged mice (46). Furthermore, adoptive combination therapy involving T cells expressing FL substantially increased host DC and T cell activation and enhanced antitumor immunity (47). Consequently, in this study, we investigated the immunomodulatory effect of co-administration of FL with the rVCG-Pmp18.1 vaccine as a strategy to enhance the protective efficacy and the potential mechanism of immunomodulation. We show that co-administration of FL adjuvant with rVCG-Pmp18.1 vaccine enhanced the cellular and humoral immune responses and protection against Cab infection. The immunomodulatory action of FL was associated with its ability to mobilize innate and adaptive immune cells into the mucosal and systemic immune inductive sites.

## Materials and Method

### Ethics Statement

In this study, the recommendations contained in the Guide for the Care and Use of Laboratory Animals of the National Institutes of Health were followed. The Institutional Animal Care and Use Committee (IACUC) of Morehouse School of Medicine (MSM) (Assurance number A3381-01) approved the study protocol (Protocol Number: 16-15). MSM-IACUC adheres to the National Institute of Health (NIH) guidelines for the care and 184 use of laboratory animals, the Public Health Service (PHS) policy, and the Animal Welfare Act.

### Reagents

*Chlamydia abortus* strains P16 and B577 (ATCC VR-656) are laboratory stocks generated by propagating elementary bodies (EBs) in BGMK cells. EBs were purified by density gradient centrifugation over renografin as reported previously (48) and stored at -70°C. Purified mouse Fms-like tyrosine kinase 3 ligand (FL) was purchased from R&D Systems, Minneapolis, MN. All mice used in these studies were of the C57BL/6J strain (female, aged 6 to 8 weeks) from The Jackson Laboratory (Bar Harbor, ME). They were housed in the animal facility of Morehouse School of Medicine and animal study protocols were performed in compliance with institutional IACUC and federal guidelines.

### Construction of Vaccine Vectors and Expression of Recombinant Proteins

The vaccine vectors, pST-Pmp18.1 and pET-Pmp18.1 expressing the N-terminal fragment of the polymorphic membrane protein, Pmp18D and subsequent purification of recombinant protein has been described (49). Protein expression was detected by SDS-PAGE and immunoblotting analysis as previously described (32) using purified rabbit anti-Pmp18D polyclonal antibody. Production of VCG expressing the vaccine antigen from pST-Pmp18.1 was by gene E-mediated lysis of the growing culture following induction of protein expression by addition of IPTG essentially as previously described (50). Lyophilized VCG preparations were stored at regular refrigeration temperature (4-8°C) until used.

### Immunization, Challenge, and Analysis of Protective Immunity

**Figure 1:**
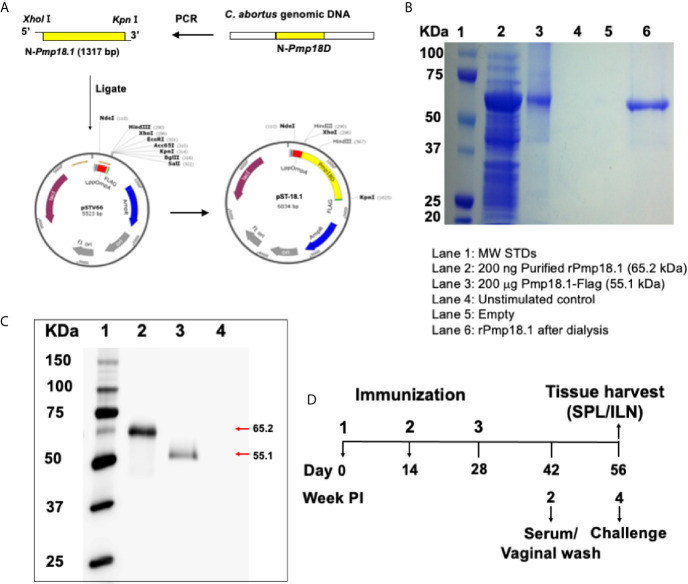
Construction of vaccine vectors and expression of recombinant proteins. **(A)** The pST-18.1 vaccine vector was constructed by genetically inserting the amplified 1317 bp fragment of the N-terminal Pmp18D coding sequence in frame with the N-terminal LppOmpA and C-terminal FLAG in plasmid vector pSTV66. The pET-18.1 expression vector was similarly constructed by inserting the 1317 bp fragment into vector pET-32a and rPmp18.1 was purified using the Ni-NTA Purification System. **(B)** Coomassie stained SDS-PAGE gel. Lane 1, MW markers; Lane 2, 200 ng of purified rPmp18.1; Lane 3, 200 µg of Pmp18.1 expressed from plasmid pST-18.1; Lane 4, Unstimulated control; Lane 5, Empty; Lane 6, PBS dialyzed rPmp18.1. **(C)** Protein expression was detected by immunoblotting analysis using purified rabbit anti-Pmp18D polyclonal antibody. Lane 1, MW markers; Lane 2, purified rPmp18.1; Lane 3, rPmp18.1-Flag fusion protein expressed from plasmid, pST-18.1; Lane 4, Empty. **(D)** Schematic diagram of the experimental design outlining the immunization, challenge, and sample collection schedules.

Groups of mice (12/group) were immunized intranasally (IN) with lyophilized rVCG-Pmp18.1 (1.5 mg) with or without FL (150 ng) or 10 μg of purified rPmp18.1 in 20 µl of PBS per mouse and boosted twice, at 2-week intervals (**Figure 1D**). Other groups were immunized with PBS alone (medium control) or 1.5 mg of lyophilized rVCG-gD2 (antigen control). A 1.5 mg dose of lyophilized rVCG-rPmp18.1 contains approximately 3 μg of purified rPmp18.1 antigen. Mice were immunized while under isoflurane anesthesia, induced with 2-4% isoflurane (Henry Schein Animal Health, Dublin, OH) in 100% oxygen in an anesthetic chamber for 30 min. Three weeks after the last booster dose, mice (6/group) were injected subcutaneously with Depo Provera (2.5 mg/mouse; UpJohn Co., Kalamazoo, MI) to synchronize the estrous cycle and facilitate a productive infection and then challenged intravaginally one week later with live *C. abortus* strain B577 (1 x 10^6^ IFUs). After challenge, mice were observed twice daily to monitor health status, such as clinical signs of adverse reaction to infection. To assess the level of infection, cervicovaginal swabs were collected from each animal every 3 days following the challenge and chlamydiae were isolated from swabs in tissue culture by standard methods (48). Experiments were repeated to contain 10-12 mice per group.

### Sample Collection

Four weeks after the last booster immunization, animals designated for immunogenicity studies (6 mice/group) were sacrificed and the spleens (51) and iliac lymph nodes (ILN) draining the genital tract were harvested. Single-cell suspensions were obtained from the tissues using the gentleMACS Dissociator (Miltenyi Biotech, Auburn, CA). Following dissociation, the tissues were filtered, centrifuged, and resuspended in PBS/BSA EDTA buffer. The Pan T Cell Isolation Kit II and the Midi magnetic bead-activated cell-sorting (MidiMACS) separator (Miltenyi Biotech, Auburn, CA) was used to purify total T cells by negative selection. A distinct pool of γ-irradiated (2000 rad) splenocytes prepared from naive animals served as a source of antigen-presenting cells (APCs). Two weeks after the 2nd and two and four weeks after the 3rd immunization, blood samples were collected by submandibular bleeding and centrifuged to obtain serum, while vaginal lavage was obtained by washing the vaginal vault with 100 µl of PBS. Samples were stored at -80 °C until analyzed.

### Assessment of Th1/Th2 Cytokines by Cytokine ELISA

The level of Pmp18.1-specific Th1 and Th2 response was assayed by measuring the antigen-specific IFN-*γ*, IL-12, IL-4 and IL-10 cytokine production by T cells isolated from spleen and ILN as previously described (30). Briefly, purified T cells were plated in quadruplicate wells of 96-well tissue culture plates at 1×10^6^ cells/well and cultured with APCs (1×10^6^/well) and 5 μg/ml of rPmp18.1. Control cultures contained APCs and T cells without antigen. After incubation for 72 h, the Bio-Plex cytokine assay kit in combination with the Bio-Plex Manager software (Bio-Rad, Hercules, CA) was used to measure cytokine concentrations in harvested supernatants. The concentration of the cytokines in each sample was obtained by extrapolation from a standard calibration curve generated simultaneously. All assays were performed in quadruplicate and were repeated for validation. The mean and SD of all quadruplicate cultures were calculated.

### Assessment of T Cell Proliferation

The ability of purified immune T cells to proliferate in response to *in vitro* restimulation in culture with Pmp18.1 was assessed using the 5-Bromo-2’-deoxy-uridine (BrdU) cell proliferation assay according to the manufacturer’s instructions (Roche Molecular Biochemicals, Indianapolis, IN) and described previously (30). Briefly, gamma-irradiated (2000 rad) splenocytes (10^6^/ml) purified from naive animals were co-cultured with purified T cells (10^6^ cells/ml) and 5 μg/ml of rPmp18.1 at 37°C in 5% CO_2_. After 3 days, the plates were incubated with BrdU labeling solution for 18 h followed by incubation with peroxidase labeled anti-BrdU antibody for 1 h at 37°C. Plates were then developed with 2,2’-azino-bis (3-ethylbenzthiazoline-6-sulfonic acid) (ABTS) substrate for 30 min and BrdU incorporation was detected using a scanning multi-well spectrophotometer (Spectra-Max 250 ELISA reader, Molecular Devices, Sunnyvale, CA). The stimulation index (SI) was calculated as the ratio between stimulated and non-stimulated cells for triplicate cultures. The experiment was repeated twice for confirmation.

### Measurement of Antibody Concentrations

A standard ELISA procedure described previously (52) was used to measure the concentration of Pmp18.1-specific antibodies (IgG, IgG2a, and IgA) in sera and vaginal washes obtained at different time points. Briefly, 96-well microtiter plates (Nunc Life Technologies, Rochester, NY) coated overnight with rPmp18.1 (2 μg/well) in PBS were blocked with 5% Non-fat dry milk (Bio-Rad, Hercules, CA) and incubated with vaginal wash or twofold serial dilutions of serum at room temperature. Following incubation with horseradish peroxidase-conjugated goat anti-mouse IgA, IgG, or IgG2c isotype (Southern Biotechnology Associates, Inc., Birmingham, Ala.), plates were developed with 3, 3’, 5, 5’-Tetramethyl Benzidine (TMB; Sigma, St Louis, MO) substrate and the absorbance was read on a Microtiter plate reader at 492 nm. The results were generated simultaneously with a standard curve, and the data sets representing the mean values from triplicate wells are shown as mean concentrations (ng/ml) ± SD.

### Immune Cell Isolation From Spleen and Lymph Nodes

In a separate experiment, spleen and ILN were harvested from mice (5/group) immunized with rVCG-Pmp18.1 with or without FL as described above, 2 weeks postimmunization. Single cell suspensions were prepared by homogenization using the gentleMACS™ Dissociator (Miltenyi Biotech, Auburn, CA) and filtration through 70-μm cell strainers (Corning Life Sciences, Edison, NJ). Splenic erythrocytes were lysed by resuspending the cell pellet in 5 ml of RBC lysis buffer (Biolegend, San Diego, CA) for 5 min. The cells were washed twice with Cell Staining Buffer, counted using a TC20 cell counter (BioRad, Hercules, CA) and used for flow cytometry.

### Flow Cytometry

Cells (1.5 x 10^6^/well) were suspended in Cell Staining Buffer (Biolegend, San Diego, CA) and stained for 30 min at 4°C with Mouse BD Fc Block (purified rat anti-mouse CD16/CD32; Clone 93) (BioLegend San Diego, CA) to reduce non-specific FcR-mediated binding. BD Horizon Fixable Viability Stain 700 (FSV700) (BD Biosciences, San Jose, CA) was then added to exclude dead cells before staining with fluorophore-conjugated antibodies. The following anti-mouse antibodies (clones) were used: anti-CD11c-PerCP-Cy5.5 (Clone N418), anti-F4/80-APC (Clone BM8), anti-CD49b-PerCP-Cy5.5 (Clone HMα2) (Biolegend, San Diego, CA), anti-MHC II-FITC (Clone 10-3.6), anti-CD68-BV421 (Clone FA/11), anti-Ly6C-BV605 (Clone AL-21), anti-CD3-BUV395 (Clone 145-2C11), anti-CD4-FITC (Clone Gk1.5), anti-CD8-APC (Clone 53-6.7), anti-CD44-PeCy7 (Clone IM7), anti-NK1.1-PE (Clone PK136), anti-TCR γ/δ-BV421 (Clone GL3) and anti-CD19-PE (Clone 1D3) (BD Biosciences, San Jose, CA). After staining, cells were washed twice with Cell Staining Buffer and fixed with 2% Paraformaldehyde for 10 min. Following an additional wash, cells were resuspended in Cell Staining Buffer and analyzed by Flow Cytometry on a BD FACSAria Fusion cell sorter in combination with BD FACSDiva software (BD Biosciences, San Jose, CA), and data was analyzed with FlowJo version 10.7.1 (BD Biosciences, San Jose, CA). Anti-mouse CompBeads (BD Biosciences, San Jose, CA) were used for compensation.

### Gating Strategy

Gates were set using the fluorescence-minus-one (FMO) gating strategy. Cells were gated first on a forward (FSC) and side scatter (SSC). Live cells were then isolated from total single cells based on the viability staining. This gating strategy allows for the selection of all live immune cells while eliminating doublets from analysis. CD4, CD8, NKT and *γ*δ positive T cells were gated from CD3 positive parent population. CD44 positive cells were gated from the CD4+/CD8+ T cell population. NK 1.1 positive NK cells, Ly6C positive monocytes, CD11c MAC II positive dendritic cells and CD68/F4/80 positive macrophages were gated from non-B and non-T cells. CD19 positive B cells were gated from the non-T cell population.

### Statistical Analysis

Statistical analyses were performed with the GraphPad Prism 9 package (GraphPad Software, Inc. La Jolla, CA, USA) on a Mac computer. Statistical differences between two groups (IFUs) were evaluated by a Two-tailed Paired t-test and between more than two groups (cytokine and antibody concentrations, T cell proliferation) by one-way ANOVA. Differences were considered to be significant at p* < 0.05.

## Results

### Generation and Expression of Vaccine Antigens

The pST-18.1 vaccine vector was constructed such that the 1,317 bp fragment of the N-terminal Pmp18D coding sequence was inserted in frame with the C-terminal FLAG contained in plasmid pSTV66 (**Figure 1A**). Sequencing results of the newly generated plasmid construct confirmed that the cloned gene fragment was in frame with FLAG. Also, the pET-18.1 expression vector was constructed to contain the same gene fragment of N-terminal Pmp18D sequence inserted into vector pET32a and rPmp18.1 was purified using the Ni-NTA Purification System. Purity of the purified protein was determined by Coomassie staining (**Figure 1B**). Following transformation of *V. cholerae* 01 or *E. coli* BL21 competent cells with plasmid pST-18.1 or pET-18.1 respectively, expression of the recombinant rPmp18.1 protein was confirmed by Western immunoblotting analysis using polyclonal antibody to Pmp18D (**Figure 1C**).

### FL Enhanced the Antigen-Specific Th1 Cytokine Response Profile Stimulated by Immunization With rVCG-Pmp18.1

**Figure 2:**
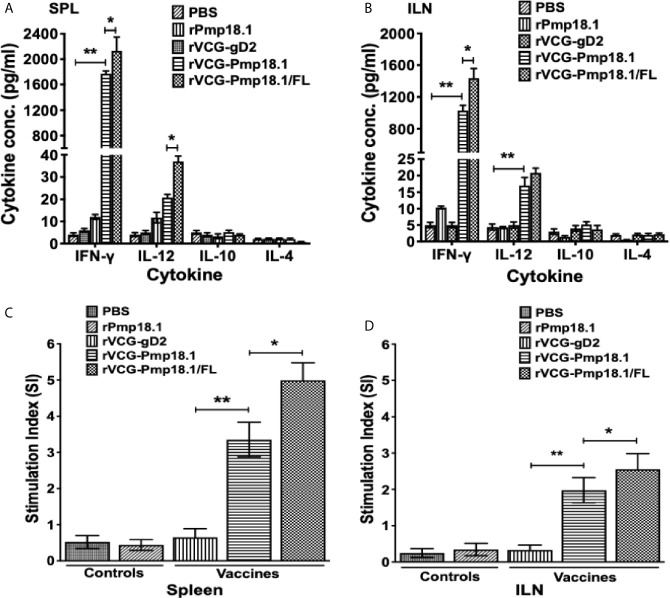
Antigen-specific genital mucosal and systemic cytokine and proliferative responses. T cells purified from the SPL and ILNs of immunized mice and controls 4 weeks postimmunization, were restimulated *in vitro* with 5 μg/ml of rPmp18.1 for 72 h. Control cultures contained APCs and T cells without antigen. The concentration of Th1/Th2 cytokines in the spleen, SPL **(A)** and ILN **(B)** contained in supernatants of culture-stimulated cells was measured using the Bio-Plex cytokine assay kit. All assays were performed in quadruplicate and were repeated for validation. Data were calculated as the mean values (± S.D.) for quadruplicate cultures for each experiment. The cultures without antigen did not contain detectable levels of cytokine and so the data were excluded from the results. The data shown is a representative of two assays with similar results. The antigen-specific proliferative responses in the SPL **(C)** and ILN **(D)** were determined 4 weeks after the last immunization using the BrdU cell proliferation assay kit. BrdU incorporation was detected by addition of anti-BrdU antibody, and the absorbance was read at 405 nm. The experiment was repeated twice for confirmation. The results are expressed as the stimulation index (SI), the ratio between absorbance values of stimulated and non-stimulated cells and the bars represent the mean and S.D. of six replicates from two independent experiments. Significant differences between groups were evaluated by One-way ANOVA with Tukey’s post multiple comparison test at p* < 0.05 and p** < 0.01.

To examine the immunomodulatory effect of FL on Th1 response induced by the vaccine, total immune T cells purified from SPL and ILN draining the genital tract of immunized mice obtained 4 weeks postimmunization (Day 56) were analyzed for specific Th1 and Th2 cytokine (IFN-*γ*, IL-4, IL-10 and IL-12) secretion upon restimulation with rPmp18.1. As expected, T cells from SPL and ILN of mice immunized with PBS or rPmp18.1 or VCG expressing the Cab irrelevant antigen, rVCG-gD2 did not generate significant levels of cytokines in response to rPmp18.1 stimulation. On the other hand, T cells from both SPL and ILN of mice immunized with rVCG-Pmp18.1 with and without FL induced high levels of IFN-γ and basal levels of IL-4 and IL-10 (**Figures 2A, B**). Significantly higher (*p*< 0.05) amounts of antigen-specific IFN-*γ* were produced by both splenic (**Figure 2A**) and ILN (**Figure 2B**) immune T cells from mice immunized with rVCG-Pmp18.1 co-delivered with FL compared to those from mice immunized with rVCG-Pmp18.1 vaccine alone. The secretion of significantly higher (*p*< 0.001) levels of IFN-*γ* compared to IL-4 by these T cells following immunization with the rVCG-Pmp18.1 vaccine indicates the induction of antigen-specific Th1 responses in both mucosal and systemic tissues. These results indicate that FL boosted the Th1 inducing ability of the vaccine.

### FL Enhanced the Antigen-Specific T Cell Activation Following Co-Delivery With the rVCG-Pmp18.1 Vaccine

The ability of immune T cells purified from the SPL and ILN of immunized mice to proliferate in response to *in vitro* restimulation in culture with rPmp18.1 was assessed by the BrdU incorporation assay. The magnitude of T cell proliferation was expressed as stimulation index (SI), defined as the ratio of the absorbance values of stimulated and non-stimulated cells. As shown in **Figures 2C, D**, mice immunized with rVCG-Pmp18.1 had significantly higher (> 5-fold higher) (p< 0.01) T cell proliferative responses in both SPL and ILN as indicated by the SI values compared to the rVCG-gD2 antigen control. Furthermore, the magnitude of splenic T cell proliferation induced by the FL adjuvanted rVCG-Pmp18.1 vaccine in both systemic and mucosal compartments was significantly higher (~ 2-fold higher) (p< 0.05) than that of the rVCG-Pmp18.1 alone. The results indicate that the T cell stimulating capacity of the rVCG-Pmp18.1 vaccine was enhanced following co-delivery with FL.

### FL Enhanced the Antigen-Specific Antibody Responses in Mice Immunized With the rVCG-Pmp18.1 Vaccine

**Figure 3:**
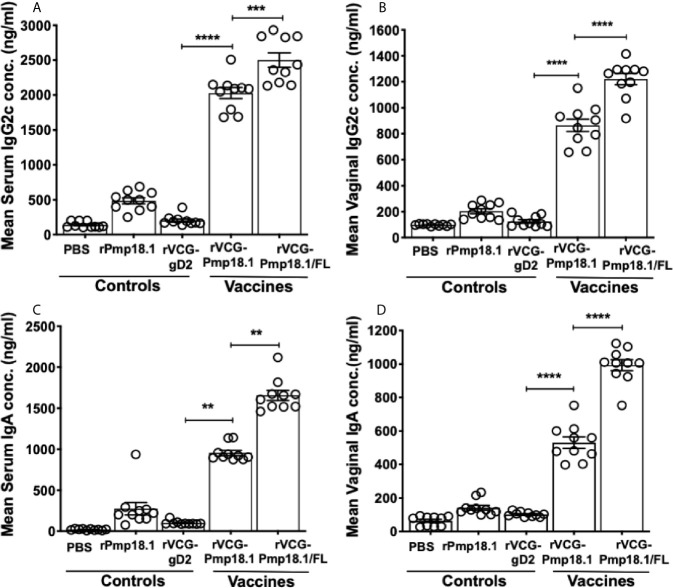
Antigen-specific IgG2c and IgA antibody responses induced in serum and vaginal wash samples. Groups of mice were immunized IN three times, 2 weeks apart. Serum and vaginal lavage samples were obtained from individual mice in each group 2 weeks after the last immunization. ELISA procedure was used to assess the concentration of IgG2c and IgA antibodies elicited in serum **(A–C)** and vaginal lavage **(B–D)** samples. The results were generated simultaneously with a standard curve and display data sets corresponding to absorbance values as mean concentrations (ng/ml) ± SD of triplicate wells for each experiment. The results are a representative of two independent experiments with similar results. Significant differences between groups were evaluated by One-way ANOVA with Tukey’s post multiple comparison test at p** < 0.01, p*** < 0.001 and p**** < 0.0001.

Specific antibody responses elicited 2 weeks after the last immunization (day 42) were measured by titrating the serum and vaginal secretions of vaccinated and control mice against rPmp18.1. **Figure 3** shows that in general, significantly higher (p < 0.0001) levels of rPmp18.1-specific IgG2c antibodies were detected in serum (A) and vaginal wash (B) of mice immunized with both vaccine formulations compared to the rVCG-gD2 control. Also, these levels were significantly higher (~2-fold higher) in serum (p < 0.001) and (~2-fold higher) in vaginal wash (p < 0.0001) of mice immunized with rVCG- Pmp18.1/FL compared to those immunized with rVCG-Pmp18.1. Similarly, immunization with rVCG-Pmp18.1 generated significantly higher (~10-fold higher) serum (p < 0.01) (C) and (5-fold) vaginal wash (p < 0.0001) (D) IgA antibody levels compared to the rVCG-gD2 control, which were increased (~10- to 19-fold higher) following co-delivery with FL. This implies that FL enhanced the magnitude of antigen-specific antibody responses elicited by the rVCG-Pmp18.1 vaccine.

### FL Enhanced the Protective Efficacy of the rVCG-Pmp18.1 Vaccine Candidate Against Heterologous Genital *C. abortus* Challenge

**Figure 4:**
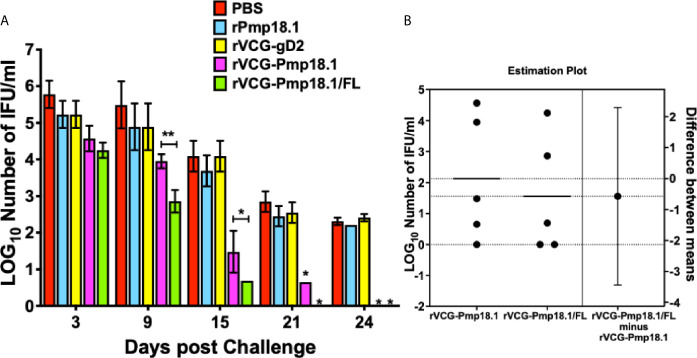
Cross protection against heterologous intravaginal challenge with *C. abortus* B577. Groups of mice immunized IN were challenged intravaginally with 1 x 10^6^ IFU of live heterologous *C. abortus* B577 4 weeks after the last immunization. Infections were monitored by cervicovaginal swabbing of individual animals at the indicated time points after infection and *C. abortus* was isolated from swabs in tissue culture and enumerated. The data show **(A)** the mean recoverable IFUs expressed as log_10_ IFU/ml ± S.D. and **(B)** the mean difference between rVCG-Pmp18.1 and rVCG-Pmp18.1/FL in an unpaired t test estimation plot. Group means at the different time points are plotted on the left axes; the mean difference is plotted on the right Y axis. The adjuvant effect size, the mean difference between means ± SEM (-0.5700 ± 1.244) is depicted as a bar. Precision of the calculated effect size as a 95% confidence interval (right axis) is indicated by the ends of the vertical error bar. Differences in the mean recoverable IFUs between rVCG-Pmp18.1 and rVCG-Pmp18.1/FL were compared by paired Student’s *t* test at p* < 0.05 and p** <0.01.

The ability of the *C. abortus* strain P16-derived vaccine candidates to protect against infection was evaluated by enumeration of genital chlamydial inclusions following intravaginal challenge of immunized mice with live *C. abortus* strain B577 four weeks after the last immunization. **Figure 4A** shows that mice immunized with rVCG-Pmp18.1 with and without FL controlled *C. abortus* replication and shedding and had shorter duration of infection compared to controls. By day 9 postimmunization, mice immunized with the rVCG-Pmp18.1/FL vaccine shed approximately 2-log lower chlamydial IFUs compared to controls and about 1-log lower IFUs compared to rVCG-Pmp18.1-immunized mice (**Figure 4A**). Furthermore, by day 21 postchallenge, rVCG-Pmp18.1/FL-immunized mice had cleared the infection while the rVCG-gD2 control-immunized mice were still shedding high numbers of IFUs. The rVCG-Pmp18.1-immunized mice shed low numbers of *C. abortus* IFUs at this timepoint, but by day 24 postchallenge, it had also completely cleared the infection. The results indicate that FL enhanced the immune effectors elicited by the vaccine to control the replication of *C. abortus*. To quantify the magnitude of the effect of the adjuvant and assess its precision on the protective immunity of the rVCG-Pmp18.1 vaccine, the two means were compared using an estimation plot (**Figure 4B**). The effect size is represented as the difference of means (0.57) at a 95% confidence interval (CI). This indicates that FL contributed to the protection of vaccinated mice.

### FL Enhanced the Mobilization of Innate and Adaptive Immune Cells to Mucosal and Systemic Tissues by the rVCG-Pmp18.1 Vaccine

**Figure 5:**
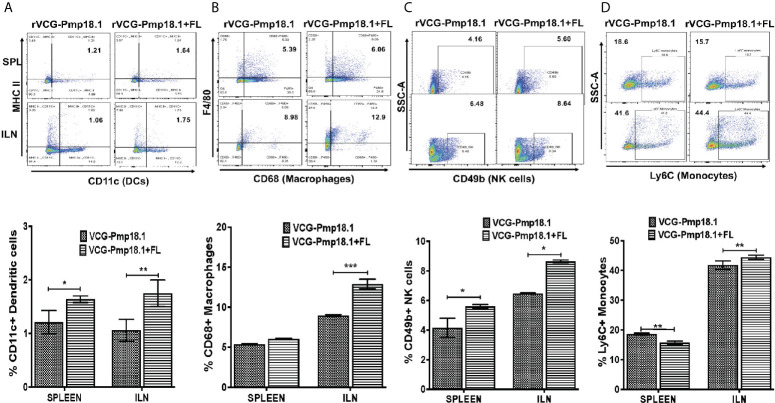
Enhanced mobilization of innate immune cells to mucosal and systemic tissues following immunization of mice with FL adjuvanted rVCG-Pmp18.1 vaccine. Groups of mice (5/group) were immunized three times IN at 2-week intervals. Four weeks postimmunization, single cell suspensions were isolated from harvested spleens and ILN and labeled for 30 min at 4°C with fluorophore-conjugated antibodies. Immune cell phenotypes and numbers were characterized by flow cytometry. Representative flow cytometric images (top) and bar graphs (bottom) showing percentages of dendritic cells **(A)**, macrophages **(B)**, NK cells **(C)** and monocytes **(D)** infiltrating the spleen and ILN after immunization. The results are shown as mean ± SD and represent two independent experiments (five mice in each group) with similar results. *p < 0.05, **p < 0.01, ***p < 0.001.

To examine the role of immune cell infiltration as a possible mechanism for Th1 enhancement by FL, we assessed the magnitude of specific innate and acquired immune cells recruited into mucosal and systemic immune inductive tissues after vaccination. For that, we isolated total cells from spleen and ILNs draining the genital tract and characterized their phenotypes and numbers following immunization with rVCG-Pmp18.1 with and without FL using flow cytometry. In general, the number of innate immune cells (expressed in percentages) were higher in the ILNs than in the spleen after immunization with rVCG-Pmp18.1 vaccine with and without FL (**Figure 5**). There were significantly higher numbers of dendritic cells (DCs) (p < 0.01) (**Figure 5A**), macrophages (p < 0.001) (**Figure 5B**), NK cells (p < 0.05) (**Figure 5C**) and monocytes (p < 0.01) (**Figure 5D**) in the ILNs of mice immunized with rVCG-Pmp18.1/FL compared to rVCG-Pmp18.1 vaccine alone. Additionally, co-delivery of FL with the vaccine significantly (p < 0.05) enhanced the percentage of DCs and NK cells in the spleens of immunized mice. However, in the spleen, the number of monocytes were significantly higher (p < 0.01) in mice immunized with rVCG-Pmp18.1 compared to those that received the FL adjuvanted vaccine. The results indicate that co-delivery of FL with vaccine boosted the infiltration of the innate immune cells important for induction of adaptive immunity, especially in the mucosal inductive sites draining the genital tract.

**Figure 6:**
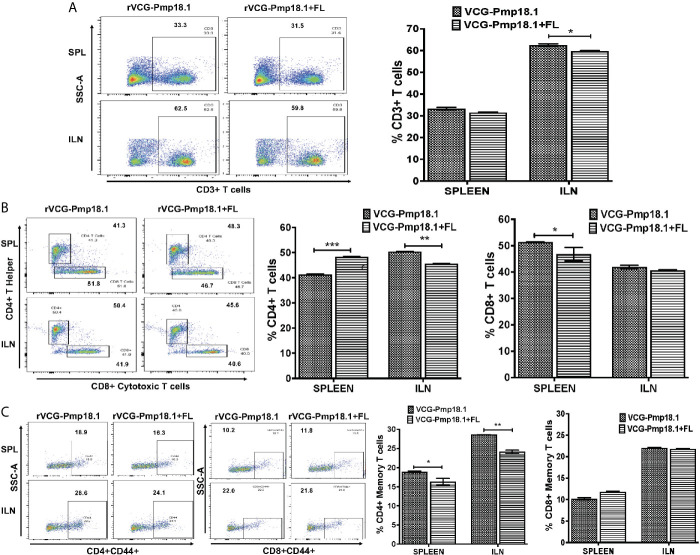
Immunization of mice with rVCG-Pmp18.1 vaccine with and without FL adjuvant induced immune CD4+ and CD8+ T cell infiltrating into mucosal and systemic tissues of immunized mice. Single cell suspensions were isolated from harvested spleens and ILN obtained from immunized mice as described above. Isolated cells were labeled with anti-CD4, -CD8, -CD4+CD44+ and –CD8+CD44+ antibodies and analyzed by flow cytometry. Representative flow cytometric images (left) and bar graphs (right) showing percentages of CD3+ T cells **(A)**, CD4+ and CD8+ T cells **(B)** and CD4+CD44+/CD8+CD44+ **(C)** memory cells. Data are shown as mean ± SD and represent two independent experiments (five mice in each group) with similar results. *p < 0.05, **p < 0.01, ***p < 0.001.

**Figure 7:**
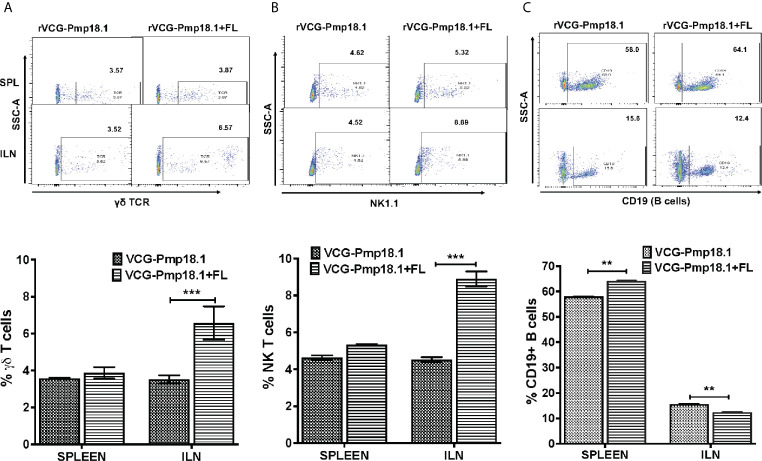
Impact of co-delivery of FL adjuvant on the *γ*δ T cells, NK T cells and B cells infiltrating the mucosal and systemic immune inductive sites of mice immunized with rVCG-Pmp18.1 vaccine. Cells were isolated from spleens and ILN of immunized mice 4 weeks postimmunization. The cells were labeled with fluorophore-conjugated anti-γδ TCR, NK1.1 and CD19 antibodies and analyzed by flow cytometry. Representative flow cytometric images (top) and bar graphs (bottom) showing percentages of *γ*δ T cells **(A)** NK T cells **(B)** and B cells **(C)** infiltrating the spleen and ILN after immunization are shown. The results are shown as mean ± SD and represent two independent experiments (five mice in each group) with similar results. **p < 0.01, ***p < 0.001.

A higher number of immune CD3+ T cells were found in the ILN compared to the spleen (**Figure 6A**). Co-delivery of FL with the vaccine significantly (p < 0.001) enhanced the percentage number of antigen specific CD4+ T cells in the spleen, but not in the ILNs (**Figure 6B**). In contrast, significantly higher (p < 0.05) numbers of CD8+ T cells were mobilized in the spleen of mice immunized with the rVCG-Pmp18.1 vaccine alone (**Figure 6B**). The number of CD4+ (CD4+CD44+) and CD8+ (CD8+CD44+) memory T cells were higher in the ILN compared to the spleen irrespective of the vaccine administered (**Figure 6C**). However, while the rVCG-Pmp18.1 vaccine induced significantly higher numbers of CD4+ memory T cells in both the spleen (p < 0.05) and ILN (p < 0.01) of immunized mice compared to rVCG-Pmp18.1/FL, comparable numbers of CD8+ memory T cells were found in both the spleen the ILNs of mice receiving either vaccine (**Figure 6C**). Furthermore, co-delivery of vaccine with FL resulted in significant (p < 0.001) increase in the percentage number of γδ (**Figure 7A**) and NK (NK1.1) (**Figure 7B**) T cells in the ILN compared to rVCG-Pmp18.1 alone. These cell numbers were comparable in the spleen following immunization with and without FL. The number of B cells were higher in the spleen compared to the ILN, irrespective of the vaccine administered (**Figure 7C**). Co-delivery of vaccine with FL resulted in a significant (p < 0.01) increase in the number of B cells infiltrating the spleen compared to vaccine alone. However, in the ILN these numbers were significantly (p < 0.01) higher following immunization of mice with rVCG-Pmp18.1 alone compared to with adjuvanted vaccine. The results indicate that varying numbers of innate and adaptive immune cells were mobilized in mucosal and systemic immune inductive sites following immunization with rVCG-Pmp18.1 in the presence and absence of FL adjuvant (**Supplementary Table 1**).

## Discussion

The current commercially available inactivated vaccines have been reported to afford marginal efficacy against infection and the live attenuated *C. abortus* vaccines cause disease leading to abortion in sheep (14). Thus, safer and cheaper efficacious vaccine alternatives would be highly desirable. Based on the shortcomings of the inactivated and live attenuated whole chlamydial vaccine candidates, the current focus is to develop vaccines based on chlamydial subunit components. As subunit vaccine candidates often require the addition of adjuvants to augment protective immunity, various adjuvants have been used to boost protective immune responses to subunit chlamydial vaccine antigens (33, 53, 54). Since DCs are important for inducing protective immunity against *Chlamydia* due to their proclivity for activating Th1 immune responses (38, 39), we examined the immunomodulatory ability of a DC function activator, the Fms-like tyrosine kinase 3-ligand (FL) when co-administered with the rVCG-Pmp18.1 vaccine as a strategy to enhance protective efficacy. Previous reports indicated that FL singly or in combination with other adjuvants enhanced the protective immunity of diverse antigens following mucosal delivery without any toxic effects (44, 46, 49, 55–57).

Immunologic evaluation revealed that IN immunization of mice with the rVCG-Pmp18.1 vaccine activated a strong local mucosal and systemic Th1-mediated immune response that was enhanced when co-administered with FL. These cellular responses were characterized by antigen-specific CD4^+^ T cells secreting high levels of IFN-*γ* and IL-12, but low levels of IL-4 and IL-10, which indicated a Th1 cytokine profile. The proliferative responses induced by the rVCG-Pmp18.1 vaccine against restimulation with rPmp18.1 antigen were similarly enhanced by FL, indicating the adjuvant effect of FL on rVCG-Pmp18.1-induced cellular immune responses. The importance of IFN−*γ*−secreting CD4^+^ T cells during *Chlamydia* infection has previously been demonstrated in both human clinical and experimental animal model studies (13, 58–60). The key role of IFN−*γ* in *Chlamydia* vaccine-induced protective immunity has also been reported (54, 61, 62).

Our results also show that IN delivery of the FL-adjuvant vaccine enhanced the local mucosal and systemic anti-chlamydial IgA and IgG2c antibody responses elicited by the rVCG-Pmp18.1 vaccine that were detected in serum and vaginal lavage of immunized mice. As in our previous studies with *C. trachomatis* immunization, higher levels of IgG2c compared to secretary IgA were elicited in vaginal secretions, highlighting the significance of the Th1-associated IgG2c antibody isotype with *Chlamydia* immunity (63, 64). While these antibodies may play a protective role against *Chlamydia*, their precise role may likely be supplementary to T cell and cytokine effector mechanisms that have been established to be crucial for chlamydial immunity. This conclusion is corroborated by studies showing that high titers of *Chlamydia*-specific antibodies, including those that neutralize chlamydial infectivity *in vitro* do not necessarily correlate with protection *in vivo* (65, 66). Accordingly, the presence of antigen-specific IgG2c and IgA neutralizing antibodies might be beneficial and contributory in an ancillary manner to controlling genital chlamydial infection (67). Among their functions, these antibodies may provide protection by blocking the initial attachment of *Chlamydia* to epithelial cells thereby limiting its spread to the upper genital tract and enhancing chlamydial clearance. Furthermore, other studies have indicated that the predominant role of antibodies in chlamydial clearance is in resistance to re-infection by enhancing rapid Th1 activation (66, 68, 69).

This study revealed that FL enhanced the ability of the rVCG-Pmp18.1 vaccine to protect mice against genital challenge with live *C. abortus* strain B577 based on its ability to reduce genital chlamydial burden and the length of *C. abortus* shedding. The specificity of vaccine efficacy of rVCG-Pmp18.1 was addressed by the inclusion of rVCG expressing the HSV-2 gD2, a *C. abortus* irrelevant antigen and PBS (carrier control). The results showed that mice immunized with rVCG-Pmp18.1 with and without FL were significantly protected from challenge, with the FL adjuvanted vaccine showing a protective advantage without any toxicity. By day 21 post challenge, mice immunized with the rVCG-Pmp18.1/FL had successfully resolved the genital challenge infection while the unadjuvanted vaccine still shed bacteria at this time point. However, mice immunized with PBS or rVCG-gD2 were not protected, indicating the antigen specificity of the protection afforded by the rVCG-Pmp18.1 +/- FL vaccines. These findings confirm our previous results indicating that FL could enhance the protective immunity induced by a VCG-based multisubunit vaccine expressing the *C*. *trachomatis* PmpD and PorB antigens following rectal mucosal and intramuscular systemic delivery without any toxic effects (49, 70). The immune stimulating ability of FL has been attributed to its propensity to target and expand DCs in mucosal and systemic tissues (46, 56) and subsequent recruitment to the immunization site (71, 72).

To investigate the mechanism of Th1 immune enhancement for *C. abortus* control by co-delivery of vaccine with FL, we tested the hypothesis that the immunomodulatory effect of FL involves the bolstering of innate and acquired immune responses at mucosal and systemic immune inductive sites following vaccination. Therefore, we compared the ability of the rVCG-Pmp18.1 vaccine in the presence and absence of FL to induce the mobilization of innate and adaptive immune cells to mucosal and systemic tissues following immunization. Enumeration of infiltrating immune cells showed that co-delivery of FL with vaccine boosted the infiltration of the innate immune cells (specifically macrophages, monocytes, dendritic and NK cells) that are important for induction of adaptive immunity, especially in the mucosal inductive sites draining the genital tract. Among the innate immune cells, DCs are the most potent APCs and are essential for the initiation of primary immune responses, specifically those critical for priming the differentiation of naïve T cells to Th1 or Th2 subsets (73, 74). DCs are highly efficient in the acquisition and presentation of antigens for stimulation of adaptive immunity, including anti-chlamydial immunity (40, 41, 75, 76) through expression of a combination of cell surface and secreted molecules that influence the type of immune response stimulated. A previous report indicated that VCG stimulated DC activation and maturation leading to enhanced chlamydial antigen presentation to immune CD4+ T cells that resulted in increased T cell proliferation and Th1-type immunity (39). Macrophages are professional antigen-presenting and proinflammatory cells (77) that were found in higher numbers in both spleen and ILN following immunization with the rVCG-Pmp18.1/FL vaccine. NK cells are effector lymphocytes of the innate immune system that control several microbial infections, including *C. abortus* by limiting their spread and subsequent tissue damage (78). While numbers of monocytes found in the spleens of mice immunized with rVCG-Pmp18.1/FL were lower compared to rVCG-Pmp18.1, they were comparably higher in the ILN draining the genial tract. Monocytes, like dendritic cells play a central role in pathogen sensing, phagocytosis, and antigen presentation to T cells (79). The finding that they constituted the highest population of APCs in the ILNs highlights their significance in antigen presentation. These results indicate that the FL adjuvant-containing vaccine enhanced the infiltration of the innate immune cells important for antigen presentation and stimulation of T cells *in vivo*, especially in the ILN draining the genital tract (mucosal tissues) following immunization. This stimulation causes T cells to proliferate, develop effector function, and subsequently differentiate into memory cells (73, 80). This suggests a cellular mobilization mechanism for the enhanced protective immunity induced by the FL-adjuvanted rVCG-based *Chlamydia abortus* vaccine.

Our study shows that IN immunization of mice induced the infiltration of high numbers of CD4+ and CD8+ effector T cells in the mucosal draining ILN and systemic splenic tissues, indicating that this route of immunization induced immune effectors according to the common mucosal and general immune system. Co-delivery of FL with vaccine enhanced the number of infiltrating CD4+ T cells in the spleen. The obligatory requirement and sufficiency of CD4+ T cells for protective immunity against *C. trachomatis* infection has been established (81). Our results showed the infiltration of higher numbers of CD4+ and CD8+ memory T cells in the ILN compared to the spleen following immunization with the rVCG-Pmp18.1 vaccine with and without FL adjuvant. Also, memory T cells of the CD8+ phenotype in the ILN were ~2-fold higher than those in the spleen. Memory CD4+ T cells play an important role in protection against subsequent chlamydial infections (82). Although in mice, CD8+ T cells are mostly associated with immunopathology (83, 84), except the evidence of partial protection in genital infection (85, 86), there is also evidence of the induction of protective CD8+ T cells by a trachoma vaccine in macaques (87). Moreover, the identification of CD8 + T cell epitopes that correlate with resolution of natural infection in humans has been reported (88). An interesting finding was the significantly higher percentage of both *γ*δ and NK T cells infiltrating the ILN following immunization with the FL adjuvanted rVCG-Pmp18.1 vaccine compared to the similar numbers infiltrating the spleen, irrespective of whether the vaccine was administered with or without FL. Previous studies reported that gamma delta T cells may play an accessory role in acquired immunity to chlamydial infection (89). NK T cells have been shown to promote Th1-type cell immunity essential for protection against primary *C. muridarum* infection through modulation of dendritic cell function (90). They also contribute to protective T cell-mediated memory immunity to chlamydial re-infection by modulating the T cell cytokine environment and inhibition of regulatory T cells (91). Lastly, we showed that the number of CD19+ B lymphocytes infiltrating the spleen was 4- to 5-fold the number in the ILN of vaccine immunized mice, and co-delivery of FL with vaccine enhanced the number of infiltrating B cells in the spleen. While the role of T cells in chlamydial immunity has long been established, a similar role for B cells has been controversial, with some studies supporting (92–97) and others contradicting the protective role of antibodies in chlamydial immunity (69, 98–100). The significance of the recent report (101) of non-antibody-dependent mechanisms of B cells in controlling primary *Chlamydia* infection in vaccine development is yet to be clarified.

In conclusion, we have demonstrated that immunization with the Cab rVCG-Pmp18.1 vaccine effectively stimulated specific mucosal and systemic immune effectors that afforded protection in mice against challenge with live *C. abortus*. Co-delivery of vaccine with FL further enhanced the immune effectors elicited by the vaccine to control *C. abortus* replication and shedding. We also showed that co-delivery of FL with vaccine boosted the infiltration of the innate immune cells important for induction of adaptive immunity, especially in the mucosal inductive sites draining the genital tract. These results indicate that the immunomodulatory effect of FL is associated with its ability to mobilize innate immune cells and subsequent activation of robust antigen-specific immune effectors in mucosal and systemic lymphoid tissues. The role of specific cell types in vaccine-mediated protection and the ability of the vaccine to protect against *C. abortus* infection-induced upper genital tract pathology, including abortion are being addressed in ongoing studies.

## Data Availability Statement

The original contributions presented in the study are included in the article/**Supplementary Material**. Further inquiries can be directed to the corresponding author.

## Ethics Statement

The animal study was reviewed and approved by The Institutional Animal Care and Use Committee (IACUC) of Morehouse School of Medicine (MSM) (Assurance number A3381-01).

## Author Contributions

FE conceived and designed the study. SR performed all the experiments. FM, TT, and SL contributed to the experimental work. FE, SR, JI, and YO contributed to analysis and interpretation of data. FE wrote the manuscript. JI, DC, and YO contributed to critical revision of the manuscript. All authors contributed to the article and approved the submitted version.

## Funding

This research was funded by the National Institutes of Health (R01AI126897) to FE. The investigation was conducted in a facility constructed with support from Research Facilities Improvement Grant #1 C06 RR18386 from the National Center for Research Resources, National Institutes of Health.

## Conflict of Interest

The authors declare that the research was conducted in the absence of any commercial or financial relationships that could be construed as a potential conflict of interest.
