# Supplementary figures and images for: Cellular Basis for the Enhanced Efficacy of the Fms-Like Tyrosine Kinase 3 Ligand (FL) Adjuvanted VCG-Based Chlamydia abortus Vaccine
Source: Front Immunol. 2021 Jun 24;12:698737. doi: 10.3389/fimmu.2021.698737 (PMC8264281; doi:10.3389/fimmu.2021.698737)

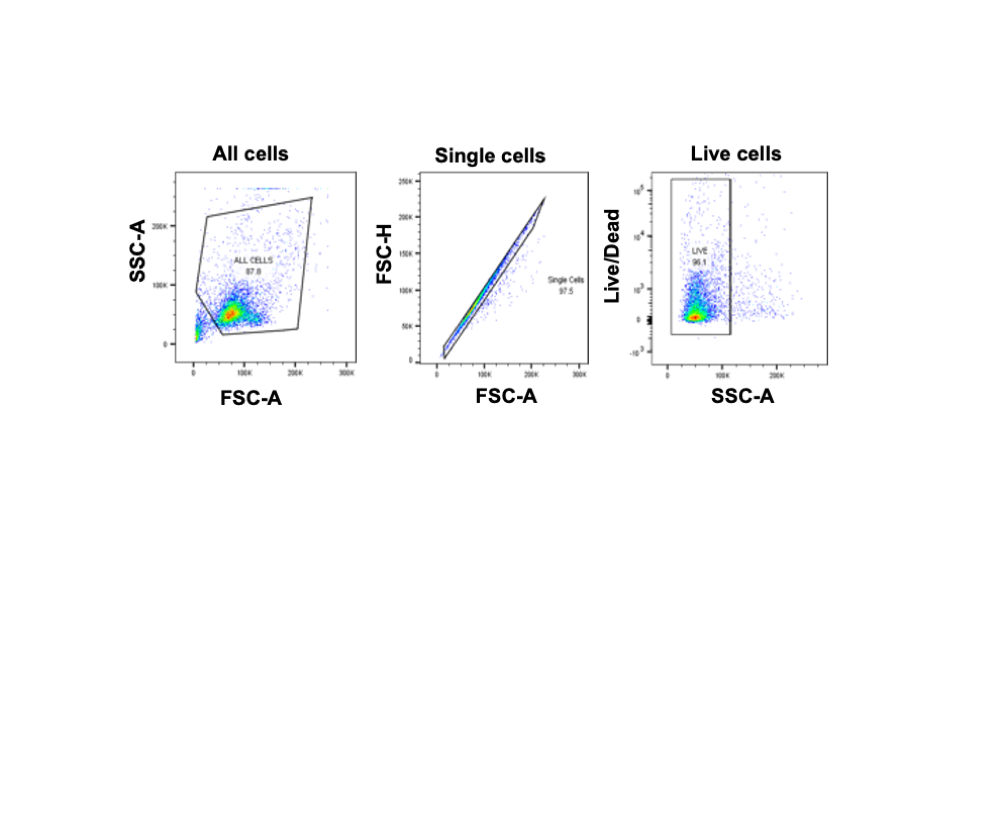

Supplement: Supplementary file 1 [file Image_1.tiff]

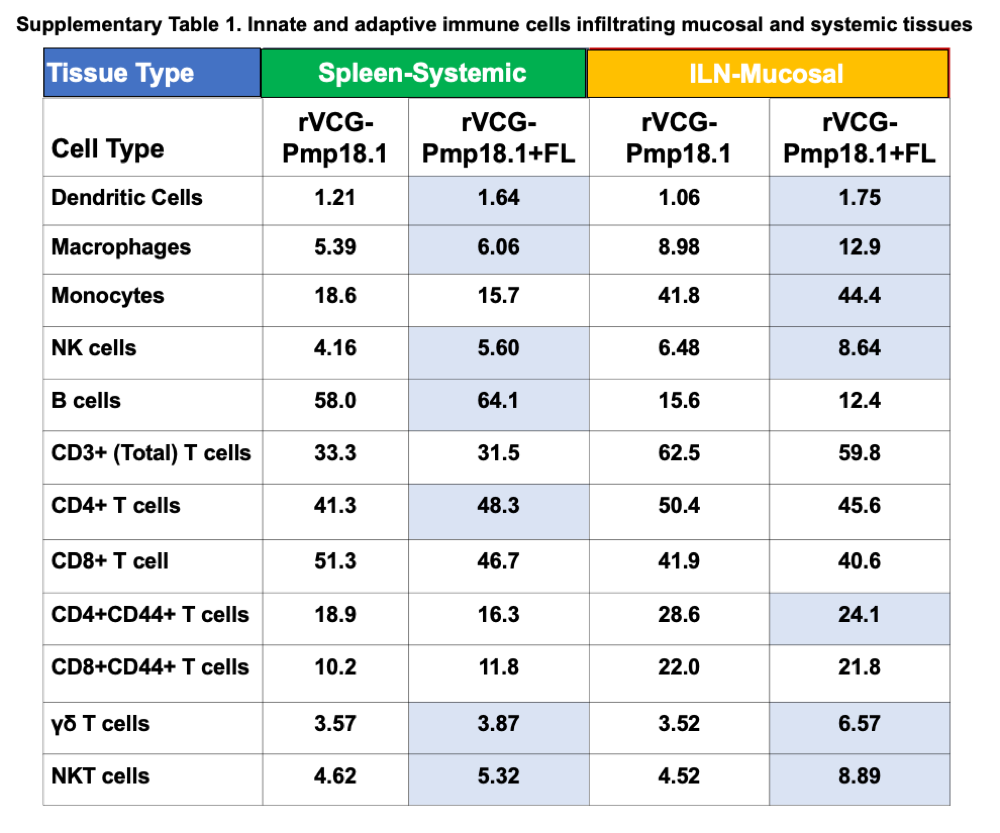

Supplement: Supplementary file 2 [file Image_2.tiff]
